# Allergic Sensitization to Perennial Allergens in Adults and Children Sensitized to Japanese Cedar or Japanese Cypress Pollen in Japan

**DOI:** 10.1155/2014/835790

**Published:** 2014-03-17

**Authors:** Masafumi Ohki, Masanobu Shinogami

**Affiliations:** ^1^Department of Otolaryngology, Saitama Medical Center, 1981 Kamoda, Kawagoe-shi, Saitama 350-8550, Japan; ^2^Department of Otolaryngology, Tokyo Metropolitan Police Hospital, 4-22-1 Nakano, Nakano-ku, Tokyo 164-8541, Japan

## Abstract

In Japan, seasonal allergic rhinitis in the spring due to exposure to Japanese cedar or Japanese cypress pollen is common. However, the allergic profile for perennial allergens in spring pollinosis remains unclear. Therefore, in this study, we investigated the allergic profiles of 652 patients with rhinitis. Total serum IgE, serum-specific IgE, and blood eosinophil counts were measured. Allergic sensitization, determined by the serum allergen-specific IgE level, did not always correspond with the patient's symptoms. Only 27% of patients with allergic symptoms in response to spring pollens were sensitized to these allergens alone; 31% of patients were also sensitized to perennial allergens, even without symptoms due to perennial allergens. Total serum IgE and eosinophil cell counts were significantly elevated in patients sensitized to perennial allergens and spring pollens, as compared to patients sensitized only to spring pollens. Most children sensitized to spring pollen (84%) were sensitized to perennial allergens, at a higher rate than adults (49%). Patients sensitized to spring pollens are likely to be latently sensitized to perennial allergens. This is especially true for children and should be monitored closely. Improvement in seasonal allergic conditions, including latent perennial allergy, is important to prevent symptoms that could advance to asthma.

## 1. Introduction

Seasonal allergic rhinitis caused by either Japanese cedar or Japanese cypress pollen is common in Japan. And Japanese cedar pollinosis is the most prevalent allergy in Japan [1]. In 2003, the prevalence of Japanese cedar pollinosis was reported to be 19.4% [2]. Such patients often complain that their symptoms occur only during the spring. However, patients may be sensitized to other allergens as well, despite being asymptomatic. Allergy is a systemic disorder that can affect the respiratory tract, eyes, skin, and gastrointestinal tract [3] and can cause asthma as well as allergic rhinitis. Perennial allergens such as mites, fungal spores, and domestic animals are considered important risk factors for the development of other allergic disorders such as asthma [4]. To control allergic diseases and prevent allergic rhinitis from progressing to other allergic disorders, it is important for patients to know their allergic profile, that is, the type and seriousness of their allergy. Therefore, we investigated the allergic profiles and sensitization to other allergens in patients sensitized to either Japanese cedar or Japanese cypress pollens.

## 2. Materials and Methods

This study was conducted on 652 consecutive outpatients (284 males, 368 females) with rhinitis, from January 2004 to March 2005. Patients enrolled were aged 2–87 years (median age, 44 years) and consisted of 544 adults above 18 years of age (225 men and 319 women; age range, 18–87 years) and 108 children below 18 years of age (59 boys and 49 girls; age range, 2–17 years). Patients with acute rhinosinusitis, common cold, and flu were excluded from the study. None of the patients had been treated previously with any specific immunotherapy. In addition to the standard otorhinolaryngological examination, we measured total serum IgE, serum-specific IgE levels (Cap-RAST (Radioallergosorbent test), Bio Medical Laboratories, Inc., Tokyo, Japan), and blood eosinophil counts. These serum tests were done at the time when rhinitis symptoms appeared. Serum-specific IgE was measured for the following allergens: Japanese cedar (*Cryptomeria japonica*), Japanese cypress (*Chamaecyparis obtusa*), grass (*Dactylis glomerata*,* Phleum pratense*, and* Ambrosia artemisiifolia*), cat dandruff, dog dandruff, molds (*Alternaria alternata*,* Aspergillus*, and* Candida*), and mites (*Dermatophagoides pteronyssinus* and* Dermatophagoides farinae*). In Japan, pollens of Japanese cedar and Japanese cypress are released mainly in spring [1]. In contrast, pollens of* Dactylis glomerata* and* Phleum pretense* peak in summer, and pollen of* Ambrosia artemisiifolia* is most prevalent in fall. These allergens were classified into three types: spring pollens, summer/fall pollens, and perennial allergens. Spring pollens included Japanese cedar and Japanese cypress. The summer/fall pollen examined was grass. Perennial allergens were cat and dog dandruff,* A. alternata*,* Aspergillus*,* Candida*, and mites. The cutoff value for serum-specific IgE levels was defined as 0.35 kUA/L. The diagnosis of Japanese cedar or Japanese cypress pollinosis is done by otorhinolaryngologist according to clinical history, nasal findings, and serum-specific IgE levels. Patients, whose serum-specific IgE levels were more than 0.35 kUA/L, were defined as having sensitization to specific allergens. Asthma is diagnosed by pulmonologists or pediatricians, according to clinical history and physical findings suggesting recurrent episodes of airflow obstruction, and spirometry to demonstrate obstruction and assess reversibility.

The statistical methods used included chi-square tests for trend assessment, the Mann-Whitney *U* test for assessments of differences between two groups. All reported *P* values were two-tailed. Statistical analyses were performed using the Ekuseru-Toukei 2012 software (Social Survey Research Information Co. Ltd., Tokyo, Japan).

This study was approved by the local ethics committee and was performed in accordance with the Helsinki Declaration (JAMA 2000; 284: 3043-3049). Informed consent was obtained from all patients or their parents.

## 3. Results

### 3.1. Sensitization in Patients Symptomatic Only to Japanese Cedar and/or Japanese Cypress

One hundred and thirty-five patients had allergic symptoms in response to only spring allergens, that is, Japanese cedar and/or Japanese cypress. Only 27% (36/135) of these patients showed allergic sensitization exclusively to these allergens, while 34% (46/135) of patients were also sensitized to perennial allergens (31%, 42/135) or summer/fall allergens (3%, 4/135) without allergic symptoms. In addition, 32% (43/135) of patients showed no sensitization to any allergen, despite the presence of allergic symptoms. Of the total, 7% (10/135) of patients were not sensitized to cedar and/or cypress pollen, but only to summer/fall pollens (1%, 2/135) or perennial allergens (6%, 8/135).

### 3.2. Allergic Sensitization in Adults and Children

Three hundred and twenty-nine (50%) of the 652 patients showed allergic sensitization in our study. One hundred and twenty-three of these patients were sensitized only to seasonal allergens (spring (18%, 115/652) or summer/fall pollen (4%, 26/652)), while 21% (137/652) of patients were sensitized to both spring pollens and perennial allergens (Table 1). Ten percent of patients (68/652) were sensitized to perennial allergens but not to spring pollens.

Two hundred and sixteen (40%) of 544 adult patients were sensitized to spring pollens. One hundred and six (49%) of 216 adults were sensitized to perennial allergens as well as spring pollens, while 43% (93/216) of adult patients showed sensitization only to spring pollens, and 8% (17/216) did only to spring and summer/fall pollen. Thirty-seven (34%) of 108 children were sensitized to spring pollens. However, only 5 of 37 children (14%) were sensitized only to spring pollens. Hence, most children (84%; 31/37) sensitized to spring pollens were also sensitized to perennial allergens.

**Figure 1:**
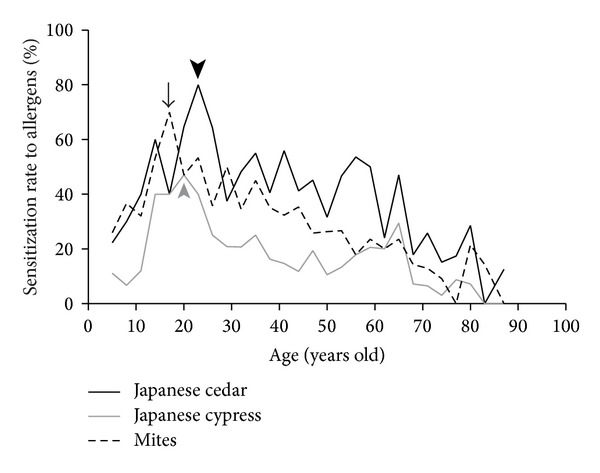
The rate of sensitization (determined by RAST) to Japanese cedar, Japanese cypress, and mites was affected by the patient's age. Black arrow head, gray arrow head, and black arrow show the peaks of each rate, respectively.

Children were significantly more likely than adults to be sensitized to perennial allergens in addition to spring pollens (*P* = 0.0001, chi-square test). The rate of sensitization to spring pollens gradually increased with the patient's age and peaked in the early 20s (Figure 1) and then fell gradually with age after that. A similar tendency was shown in perennial allergens (mites) although the rate of sensitization peaked at ages 15–17, unlike with spring pollens. In children aged 15–17, the rate of sensitization to perennial allergens (mites) was higher than it was to spring allergens. After patients reached their 20s, the rate of sensitization to spring pollens was higher than perennial allergens (mites).

### 3.3. Total Serum IgE

**Figure 2:**
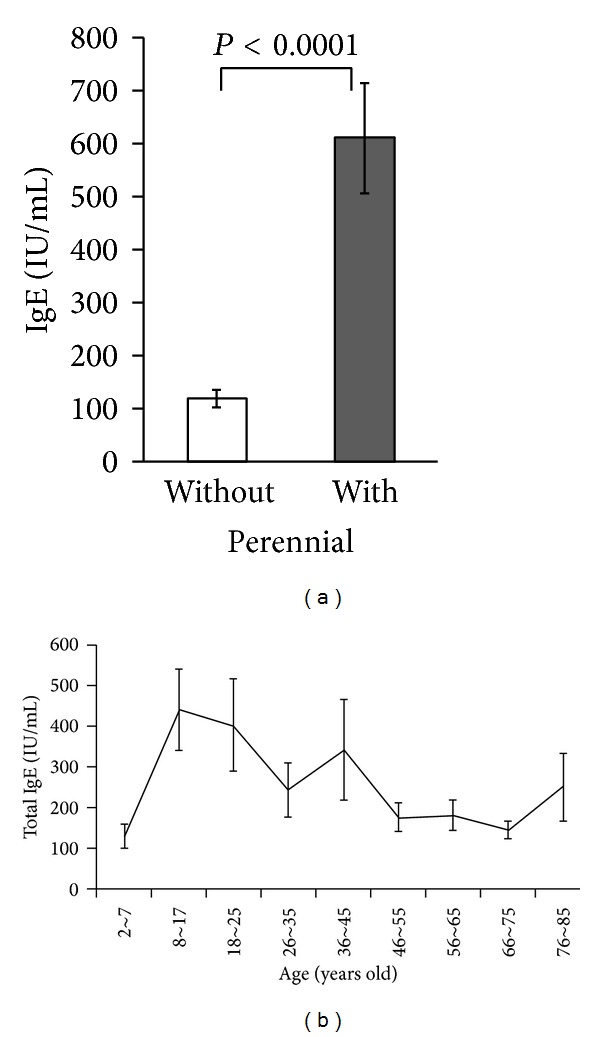
(a) Total serum IgE levels were significantly elevated in 137 patients sensitized to both perennial allergens and spring pollens (609 ± 104 IU/mL) compared to 98 patients sensitized only to spring pollens (118 ± 16 IU/mL; *P* < 0.0001, Mann-Whitney *U* test). (b) Variation of eosinophil cell count with age. This parameter was highest in children 9–17 years of age.

**Figure 3:**
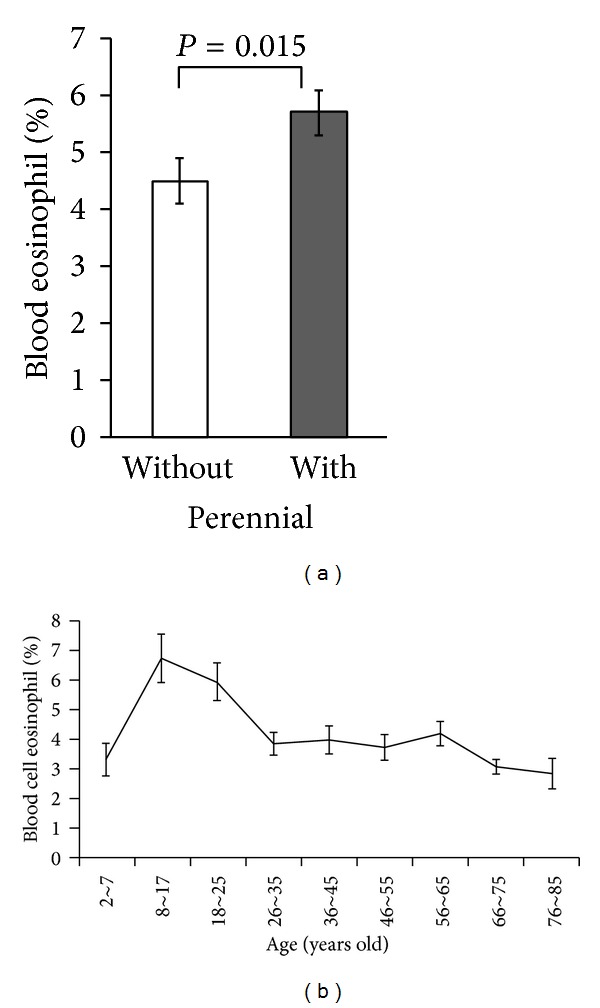
(a) Eosinophil cell count was significantly higher in patients sensitized to both perennial allergens and spring pollens (5.7% ± 0.4%) than in patients sensitized only to spring pollens (4.5% ± 0.4%; *P* = 0.146, Mann-Whitney *U* test). (b) Variation of serum total IgE levels with age. This parameter was highest in children 9–17 years of age.

The mean total serum IgE level ± S.E. was 118 ± 16 IU/mL in the 98 patients sensitized only to spring pollen and 609 ± 104 IU/mL in the 137 patients sensitized to both perennial allergens and spring pollens (Table 2). Mean total serum IgE level was significantly elevated in patients sensitized to both perennial allergens and spring pollens, as compared to patients sensitized only to spring pollens (*P* < 0.0001, Mann-Whitney *U* test) (Figure 2(a)). The average of total serum IgE levels was highest in 8-17-year olds and decreased with age (Figure 3(a)).

### 3.4. Blood Cell Eosinophil Count

The blood cell eosinophil count was also compared between groups. The eosinophil cell proportion was 4.5 ± 0.4% in patients sensitized only to spring pollens, while it was significantly higher (5.7 ± 0.4%) in patients sensitized to both perennial allergens and spring pollens (*P* = 0.0146, Mann-Whitney *U* test) (Figure 2(b), Table 2). The blood cell eosinophil count showed the same reductive tendency (Figure 3(b)).

### 3.5. Allergic Sensitization in Asthma

Fifty-nine patients (46 adults, 13 children) had been previously diagnosed with asthma. The remaining 593 patients had not been diagnosed with asthma. Sensitization to any allergen was detected in 58% of patients with asthma (34/59). Twenty-six (44%) of 59 patients were sensitized to spring pollens (Table 3). Approximately half of the asthma patients (51%; 30/59) were sensitized to perennial allergens. Seven percent of patients with asthma (4/59) were sensitized only to spring pollen, while 16% (94/593) in patients without asthma were sensitized exclusively to these allergens. Thirty-seven percent of patients with a previous asthma diagnosis (22/59) were sensitized to both spring and perennial allergens, which was significantly higher than that observed in patients without asthma (20%; 117/593) (*P* = 0.0017, chi-square test).

Mean total serum IgE levels in patients with asthma were 477 ± 89 IU/mL, while those in patients without asthma were 224 ± 27 IU/mL (*P* = 0.0001 compared to patients with asthma, Mann-Whitney *U* test). Blood eosinophil cell proportion in patients with asthma was 5.4 ± 0.6%. In patients without asthma, the proportion was 3.9 ± 0.2%. Blood eosinophil cell proportion in patients with asthma was significantly higher than those in patients without asthma (*P* = 0.008, Mann-Whitney *U* test).

## 4. Discussion

Allergic sensitization, as diagnosed by the serum allergen-specific IgE level, does not always correspond with the patient's symptoms. We found that approximately twice as many patients were sensitized to both spring pollens and perennial allergens compared to patients sensitized only to spring pollens. However, many patients were asymptomatic to perennial allergens. Exposure to perennial allergens, such as house dust mite and cat and dog dandruff, is an important predisposing risk factor for asthma [4]. Previous diagnosis of asthma was largely related to serum IgE levels and blood eosinophil counts [5–7]. Even in nonasthmatic patients, airway responsiveness (assessed using methacholine [8]) is increased in some cases of allergic rhinitis, indicating an increased risk for asthma [9–11]. Sensitization to cat dandruff, dust mite, cockroach, and ragweed is an important predictor of airway hyperresponsiveness [12]. Airway hyperresponsiveness is strongly related to elevated total serum IgE levels, even in asymptomatic patients [5, 13]. In other words, total serum IgE level is considered an indicator of probable airway hyperresponsiveness or asthma. In our study, total serum IgE levels and blood cell eosinophil counts were significantly elevated in patients sensitized to both spring pollens and perennial allergens, as compared to patients sensitized only to spring pollens. Therefore, patients sensitized to both spring pollens and perennial allergens might be at greater risk of developing airway hyperresponsiveness or asthma.

Compared to adults, fewer children were sensitized only to spring pollens. Most children (approximately 80%) had perennial allergen sensitization as well as spring pollen sensitization if exposed previously, whereas for adults, the proportion was approximately 50%. Early childhood is a critical factor for determining whether a child will be predisposed to asthma later in life [4, 13, 14]. Sensitization to perennial allergens early in life can lead to chronic asthma, characterized by airway hyperresponsiveness and impairment of lung function [14, 15]. In addition, exposure to high levels of perennial allergens aggravates this process, while sensitization to seasonal allergens has not been shown to be important [14]. Therefore, patients with allergic rhinitis caused by spring pollen include those at high risk for developing asthma, since approximately two-thirds of individuals are concomitantly sensitized to perennial allergens without symptoms. Uncontrolled allergic rhinitis may cause the condition of patients with coexisting asthma to deteriorate [16]. On the other hand, seasonal allergens may affect asthma to some degree, considering that patients with hay fever demonstrate airway hyperresponsiveness during pollen season [10]. In addition, disease control by pollen immunotherapy could help prevent the development of asthma in children with seasonal allergic rhinitis [17]. Improvement in seasonal allergic conditions, including covert perennial allergy, is important for preventing the progression of allergic rhinitis to asthma. In particular, children with seasonal allergic rhinitis should avoid exposure to perennial allergens as well as spring pollens to prevent them from developing asthma. As we found in this study, the majority of children with seasonal allergic rhinitis are also sensitized to perennial allergens. Even children who are not allergic may benefit from minimal exposure to perennial allergens to prevent sensitization. Improvement of allergic conditions is considered important to prevent airway hyperresponsiveness and the development of asthma.

## 5. Conclusion

Patients who have been sensitized to spring pollen are also likely to have been covertly sensitized to perennial allergens. This is especially true for children. Improvement in the seasonal allergic conditions, including covert perennial allergy, is considered important for preventing the development of asthma.

## Figures and Tables

**Table 1:** Allergic sensitization.

	Adults	Children	Total
Only spring pollens	93	5	98
Only fall pollens	7	1	8
Only perennial allergens	51	15	66
Spring and fall pollens	17	1	18
Spring pollens and perennial allergens	59	16	75
Fall pollens and perennial allergens	2	0	2
Spring and fall pollens and perennial allergens	47	15	62
No sensitization	268	55	323

**Table 2:** Serum total IgE and blood cell eosinophil.

	Total IgE (lU/mL)	Eosinophil cell proportion (%)
Only spring pollens	118 ± 16	4.5 ± 0.4
Only fall pollens	172 ± 93	3.7 ± 1.4
Only perennial allergens	288 ± 51	3.2 ± 0.4
Spring and fall pollens	174 ± 30	5.2 ± 0.9
Spring pollens and perennial allergens	391 ± 67	5.4 ± 0.5
Fall pollens and perennial allergens	—	—
Spring and fall pollens and perennial allergens	878 ± 213	6.1 ± 0.6
No sensitization	120 ± 15	3.1 ± 0.2

**Table 3:** Allergic sensitization in asthma.

Only spring pollens	4
Only fall pollens	0
Only perennial allergens	7
Spring and fall pollens	0
Spring pollens and perennial allergens	14
Fall pollens and perennial allergens	1
Spring and fall pollens and perennial allergens	8
No sensitization	25
